# The Genetic Landscape of Inherited Retinal Diseases in the Israeli Population

**DOI:** 10.1167/iovs.67.4.24

**Published:** 2026-04-13

**Authors:** Sapir Shalom, Libe Gradstein, Eran Pras, Johanna Valensi, Ohad S. Birk, Anat Blumenfeld, Avital Eilat, Michal Macarov, Tomer Poleg, Frans P. M. Cremers, Susanne Roosing, Daan M. Panneman, Nadin Hollander, Nitza Goldenberg-Cohen, Claudia Yahalom, Eyal Banin, Tamar Ben-Yosef, Dror Sharon

**Affiliations:** 1 Department of Ophthalmology, Hadassah Medical Center, Faculty of Medicine, The Hebrew University of Jerusalem, Jerusalem; 2 Department of Military Medicine and “Tzameret,” Faculty of Medicine, Hebrew University of Jerusalem and Medical Corps, Israel Defense Forces, Jerusalem, Israel; 3 Department of Ophthalmology, Soroka Medical Center and Clalit Health Services, Faculty of Health Sciences, Ben-Gurion University, Beer Sheva, Israel; 4 Department of ophthalmology, Shamir Medical Center, Faculty of Medicine, Tel Aviv University, Tel Aviv, Israel; 5 The Morris Kahn Laboratory of Human Genetics, Faculty of Health Sciences, Ben Gurion University, Beer Sheva, Israel; 6 Genetics Institute at Soroka Medical Center, Beer Sheva, Israel; 7 Danek Gertner Institute of Human Genetics at Sheba Medical Center, Ramat Gan, Israel; 8 Department of Human Genetics, Radboud University Medical Center, Nijmegen, The Netherlands; 9 The “Lirot” Association, Tel Aviv, Israel; 10 Department of Ophthalmology, Bnai-Zion Medical Center, Haifa, Israel; 11 Ruth and Bruce Rappaport Faculty of Medicine, Technion–Israel Institute of Technology, Haifa, Israel

**Keywords:** genetic landscape, IRDs, founder variants, ethnicity

## Abstract

**Purpose:**

Inherited retinal diseases (IRDs) are a group of more than 50 clinically and genetically heterogeneous diseases caused by variants in more than 300 genes. The Israeli population is composed of multiple ethnic groups with variable prevalence of IRD-causing variants. In the current study, we analyzed IRDs in different Israeli ethnic groups to establish the genetic landscape.

**Methods:**

Patients were recruited by five genetic centers, and eight ophthalmic centers, located throughout the country, and belonging to the Israeli inherited retinal disease consortium (IIRDC). The information regarding the cause of disease in each solved family was tabulated. For each ethnic group, we listed the causing variants and their frequencies.

**Results:**

We identified a total of 1062 disease-causing variants in Israeli patients with IRDs from 20 ethnic groups (13 of which are Jewish), with a total of 4,728 familial pathogenic alleles. Founder variants contributed the largest proportion of alleles in Yemenite Jews (75%), followed by Turkish Jews (67%), and North African Jews (66%). The most common disease-causing variant was *ABCA4*-c.5882G>A, a pan-ethnic variant, followed by *FAM161A*-c.1355_1356del, a founder variant in multiple Jewish ethnic groups. By performing haplotype analysis, 21 additional founder variants were identified. We generated a searchable online database (https://www.eyes.org.il/genecal) based on this data depicting the most common variants for each ethnic group and IRD.

**Conclusions:**

Our analysis provides a comprehensive list of common and founder variants for each ethnic group in Israel and is likely to allow more accurate and informative genetic counseling for Israeli families with IRDs.

The Israeli population has a unique structure, includes multiple ethnic groups, and can be divided into Jews (74%), Arab-Muslims (18%), Bedouins (3%) Druze (2%), Arab-Christians (2%) and others (1%) (according to the central bureau of statistic, December 2022). The various Jewish ethnic groups share an ancient genetic background that was disrupted by exile out of their homeland, settling in different and sometimes isolated geographic areas, resulting in founder variants that are specific to each ethnic group.^1^^–^^4^ The Arab-Muslim, Druze, and Bedouin sub-populations usually live in villages that originated from a small number of founders and show relatively high rates of consanguinity that result in village-specific common founder variants.^4^^–^^6^

This unique population structure with a relatively high rate of founder variants and consanguinity results in a high prevalence of recessive diseases. For example, Tay-Sach is highly prevalent among Ashkenazi Jews because of a founder variant in *HEXA*.^7^ Similarly, familial Mediterranean fever is also prevalent among Jews with a shared haplotype in Moroccans Jews.^8^ Other sub-populations in Israel also share founder variants such as p.Glu452del in *MUTYH* causing familial adenomatous polyposis detected in Arab-Muslims and Druze with a shared haplotype.^9^

Inherited retinal diseases (IRDs) are a group of more than 50 phenotypes that cause visual impairment because of photoreceptor or retinal pigmented epithelium (RPE) degeneration/dysfunction.^10^^,^^11^ The worldwide prevalence of IRDs is estimated at ∼1:3400 individuals, with high variability between different populations.^12^ The most common IRD type is retinitis pigmentosa (RP) with a worldwide prevalence of 1:4,660,^12^ characterized by a progressive loss of rod followed by cone photoreceptors.^13^^,^^14^ IRDs are highly heterogenous genetic disorders which can be caused by variants in about 300 genes that can be inherited in all modes.^11^^,^^15^

The prevalence of IRDs in the Israeli population is relatively high, similar to other diseases with an AR component, and estimated as ∼1:1,000 due to consanguinity and marriages within the community.^16^ The segregation of ethnic groups and subpopulations in Israel leads to common founder variants such as the *CERKL* founder variant in Yemenite Jews^17^ or founder variants in *MAK* and *DHDDS* in the Ashkenazi Jewish*.*^18^

In this work we aimed to better characterize the ethnic-related genetic landscape of IRDs in Israel. Nationwide data, as well as the disease-causing variant and phenotypic distributions, were compared among different ethnic groups. The results were further compared to the general Israeli population and worldwide data.

## Methods

### Study Design

This multicenter study was carried out by members of the Israeli inherited retinal disease consortium (IIRDC)^19^ with assistance of the Lirot association, a nonprofit organization (https://www.eyes.org.il/Home). Genetic data, some of which were previously published in a different form,^19^ were collected from five IIRDC genetic centers and eight clinical ophthalmic centers throughout the country. The data included all cases from previous study^19^ plus additional patients tested since. Clinical and molecular diagnosis of IRDs in affected individuals was performed in the participating IIRDC centers as described earlier.^19^ The following details were collected on index cases from each family: IRD phenotype, ethnic origin, inheritance pattern, presence of consanguinity in the family, causative gene and variant(s). Ethical approval was obtained from the Institutional Review Boards of participating centers. The tenets of the Declaration of Helsinki were followed. Participants provided written informed consent after receiving an explanation about the study and its possible consequences.

### Genetic Analysis

Genetic analysis was performed in each IIRDC center and included mainly whole exome sequencing (WES) and panel sequencing. Variants files were annotated using the Franklin (https://franklin.genoox.com/clinical-db/home) platform, and the data was filtered using an IRD gene panel. Variants were considered disease-causing according to ClinVar photogenic/likely pathogenic classifications. Variants of uncertainty significance or variants with no previous classification were considered disease-causing if they were common in our patient cohort, had functional predictions, were in trans with null variants.

### Population Analysis

Demographic information was collected from the Israeli central bureau of statistics (https://www.cbs.gov.il/en/cbsNewBrand/Pages/default.aspx). Information regarding the number of Jewish and other ethnic groups was set to September 2023. Information on the number of Bedouin participants was collected from the Ben Gurion University database and was set to June 2023 (https://in.bgu.ac.il/humsos/negevSus/SYBSN/Pages/default.aspx). The calculation of the enrichment score is in the supplementary methods.

### Pan-Ethnic Analysis

Pan-ethnic variants refer to those that can be detected in multiple populations. Such variants can either arise from mutational hotspots or from ancient alleles that are spread throughout various populations. We calculated a population-based score using data from the gnomAD database (hg38, version 4.1.0) (https://gnomad.broadinstitute.org/) aiming to assess whether a variant is pan-ethnic. To this end, only variants with at least 10 familial alleles in the study cohort were used for this analysis. The five major gnomAD populations were included: African (African/African American), United States (Admixed American), European (European [non-Finnish]), East Asia and South Asia. For each population, the minor allele frequency of each variant was calculated by dividing the allele count by the allele number. A variant was considered pan-ethnic if it was present in at least three out of the five gnomAD populations. Some of the pan-ethnic pathogenic variants were also enriched in a specific ethnic group and we term them as “Pan-ethnic population enriched” (PPE) if their frequency was more than 50% in a single ethnic group.

### Founder Variants Analysis

Disease-causing variants with 10 or more index case alleles were suspected as founder and selected for haplotype analysis. Haplotype analysis of molecular inversion probes (MIPs)^20^ or WES data was performed using the Franklin platform (https://franklin.genoox.com/clinical-db/home). Information regarding the ethnic origin, consanguinity, and sequencing method were collected. MIPs samples were analyzed for variants only in a specific gene, whereas for WES samples the analysis was extended upstream and downstream of each gene. Disease-causing variants with either MIPs or WES data for only one patient or with low-quality variants were excluded from the analysis. The genotype of the relevant list of variants was tabulated for all cases in a single excel sheet. Some variants were also confirmed using IGV (https://igv.org/app/), as needed.

### Online Gene Calculator Tool

A gene calculator tool for presentation of the data was developed by the CATOM company (https://www.catom.com/Home).

## Results

Aiming to better characterize the ethnic-related genetic landscape for IRDs in Israel, we collected genetic information of 2676 Israeli index cases. Overall, we collected information on 1062 disease-causing variants (Supplementary Table S1), with a total of 4728 familial alleles in 202 IRD-associated genes. In-line with our previous publication,^19^ the most common causative genes are *ABCA4* (15%), *USH2A* (6%), *FAM161A* (5%), *CNGA3* (4%) and *EYS* (4%) (Supplementary Fig. S1A), and the most common disease-causing variants across all ethnicities are *ABCA4*-c.5882G>A (4%) followed by *FAM161A*-c.1355_1356del (4%), *MAK*-c.1297_1298ins353 (3%) and *DHDDS*-c.124A>G (1%) (Supplementary Fig. S1B).

### Comparison Between Ethnic Groups

The Israeli population includes many ethnic groups that differ in their consanguinity rates and impact of founder variants. To this end, we compared the IRD genetic landscape of the various ethnic groups. The studied cohort included patients who belong to 20 different ethnic groups, 13 of which are Jewish groups. The most common groups in our cohort were Arab-Muslim (28%), Ashkenazi Jews (22%) and North-African Jews (16%). The rate of consanguinity varied between different ethnic groups with an average of 36% in our cohort: Bedouin (84%), Arab-Muslim (70%), Arab-Christian (62%), and Druze (59%) (Supplementary Fig. S2). The average number of genetically solved cases is 67% (Supplementary Fig. S3). The most common inheritance pattern is AR (82%) followed by autosomal dominant (AD) and X-linked, with different ethnic groups showing variable percentages. The rate of AR inheritance ranged from 43%–98%, whereas AD inheritance is the most common in Christians (38%) and does not exist at all among Ethiopian and Syrian Jews (Supplementary Figs. S4A, S4B). Homozygosity is the most common zygosity pattern (63%) followed by compound heterozygosity (18%) and heterozygosity for dominant alleles. Homozygosity is most common among Bedouin and Muslims (80%) and compound heterozygosity is most common among Ethiopian Jews (45%) (Supplementary Figs. S4C, S4D).

**Figure 1.**
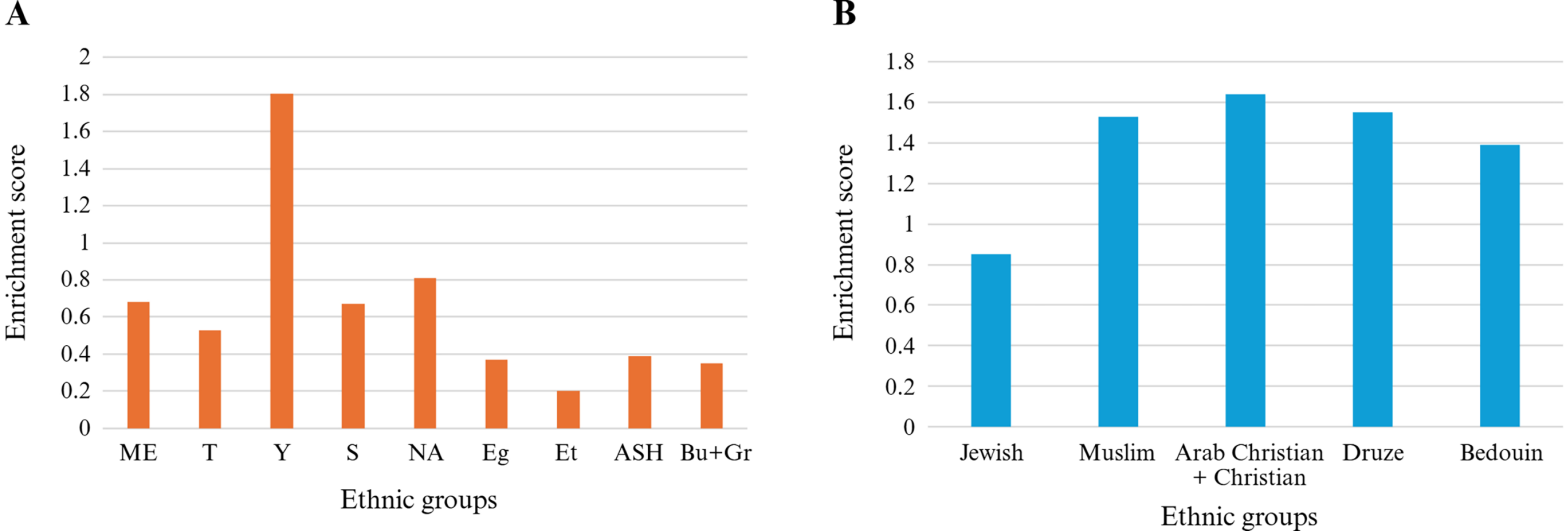
Enrichment score analysis of the IIRDC cohort in Jewish ethnic groups **(A)** and in all ethnic groups **(B)**. The score was calculated by dividing the percentage of each ethnic group in the cohort by the percentage of the same group in the general population. ME, Middle Eastern; T, Turkey; Y, Yemen; S, Syria; NA, North African; Eg, Egypt; Et, Ethiopia; ASH, Ashkenazi; Bu + Gr, Bulgaria + Greece.

We subsequently calculated the enrichment score of each ethnic group in the study cohort compared to the entire Israeli population. A score >1 indicates that the number of affected individuals in the cohort is above the expected value based on the fraction of this ethnic group in the population. The analysis revealed that among the Jewish ethnicities (Fig. 1A), only one group, Yemenite Jews, showed a high score of 1.8 indicating a relatively high representation in our cohort. Interestingly, the lowest score (0.2) was found in Ethiopian Jews, known to avoid consanguinity for at least seven generations, as we reported previously.^21^ Indeed, the consanguinity rate in this ethnic group was only 6%. On the other hand, all studied non-Jewish groups showed over-representation with enrichment scores ranging from 1.4 to 1.6 (Fig. 1B), as expected from their consanguinity rates (Supplementary Fig. S2).

**Figure 2.**
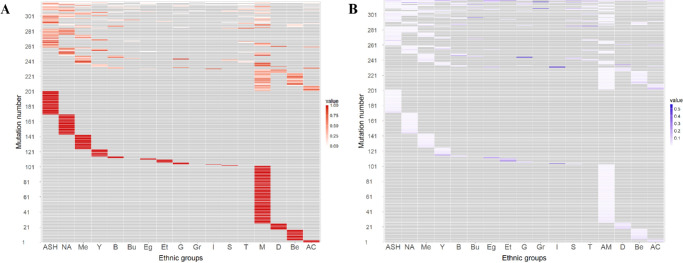
The distribution of different variants in the various populations for the 319 most common variants (retaining only disease-causing variants observed in three or more index cases). Shared variants are shown at the top of the heatmap. **(A)** Heatmap showing the ratio between 0 and 1, calculated by dividing the number of affected individuals by each variant from each origin by the total number of affected individuals for each disease-causing variant. Ratios are represented in shades of gray (0) to deep red (increasing ratio; see Supplementary Table S2 for the full list of variants). **(B)** Heatmap showing the ratio between 0 and 1, calculated by dividing the number of affected individuals carrying each variant from each origin by the total number of affected individuals from that same origin. A ratio of 0 indicates that no affected individuals from this origin carry the disease-causing variant, whereas 1 means that all the affected individuals from this specific origin carry the same variant. Ratios are represented in shades of *gray* (0) to deep *purple* (increasing ratio; see Supplementary Table S3 for the full list of variants).

To better visualize these patterns, we generated two heatmaps (Fig. 2). These heatmaps help highlight unique distributional patterns of specific variants across populations. In Figure 2A (variant-based), disease-causing variants are grouped by population of origin. Among the 319 disease-causing variants (Supplementary Tables S2, S3), 201 scored a value of 1, indicating that all index cases with each variant share the same origin. This suggests that most of the frequent disease-causing variants are population-specific. The three most prevalent groups contribute the largest share of such disease-causing variants: Arab Muslims (*n* = 77 variants), Ashkenazi Jews (*n* = 31), and North African Jews (*n* = 27). On the other hand, 35 disease-causing variants were observed in three or more origin groups. Interestingly, some of these disease-causing variants, appear to be more prevalent in specific groups (e.g., *ABCA4*-c.5882G>A, variant 319 in Fig 2A), is highly frequent among Yemenite and Ashkenazi Jews; *FAM161A*-c.1355_1356del, is highly prevalent among North African Jews). In Figure 2B (origin-based), a different perspective is presented, *FAM161A*-c.1355_1356del is the most frequent disease-causing variant in the North African Jewish group (28%, variant 316 in Fig. 2B). In contrast, the Arab-Muslim group does not show a single prevalent disease-causing variant, with the highest variant frequency reaching only 4.5%.

### Pan-Ethnic Variants

Pan-ethnic variants are defined as common variants across different populations worldwide. To define which disease-causing variants are likely pan-ethnic, we used gnomAD by comparing the five major populations and selected 28 that appeared in at least three of the populations, nine of which appeared in all five populations. One of these disease-causing variants, *ABCA4*-c.5882G>A, is known to be the most common *ABCA4* variants in multiple populations.^11^ Some disease-causing variants, such as structural variants or the exon 3 haplotype in the *OPN1LW- OPN1MW* gene cluster, could not be analyzed as candidates for pan-ethnicity using gnomAD.

We further compared the information regarding the likely pan-ethnic disease-causing variants to their distribution in the analyzed IRD cohort and identified pan-ethnic variants that have high prevalence in a specific ethnic group. For example, *USH2A*-c.802G>A appeared in all five gnomAD populations, was present in our database in only one population, Arab-Muslims. This comparison led us to determine another definition—PPE—for variants that can be considered as pan ethnic but have relatively high prevalence in a specific population. We defined disease-causing variants as PPE if more than half of the cases in the cohort belong to a single ethnic group. Out of 28 likely pan-ethnic variants, 27 were considered as PPE. The only disease-causing variant that did not fit to this definition is *ABCA4*-c.5882G>A.

### Founder Variants

As mentioned above, each ethnic group in the Israeli population differs by the rate and impact of founder variants. This raised a need to develop a set of rules that will aid categorizing the various disease-causing variants by their likelihood to be considered as founder. Using haplotype analysis, 19 founder variants were reported in the Israeli population thus far (Supplementary Table S1). Aiming to identify additional candidate founder variants, we focused on those with at least 10 familial alleles. The analysis revealed 72 disease-causing variants, with sufficient NGS data for 21 of these variants. The analysis revealed that all 21 studied disease-causing variants are indeed founder. The remaining 52 disease-causing variants were therefore considered as likely founder variants (Supplementary Table S1).

The distribution of the 40 founder variants is shown in Supplementary Fig. S5. Some genes such as *ABCA4* and *USH2A* have a relatively high number of founder variants across multiple origins, whereas others (e.g., *CERKL* and *EYS*) have only one founder variant in a specific ethnic group. Moreover, this figure highlights founder variants that are shared between all Jewish ethnic groups and shared variants between different origins such as *USH2A*-c.236_239dup in Arab-Muslims and Middle Eastern Jews.

As for the remaining disease-causing variants, we separated between two definitions: recurrent and private variants. Recurrent variants were defined as more than two familial alleles (*n* = 229) and private variants as one or two familial alleles (*n* = 750).

**Figure 3.**
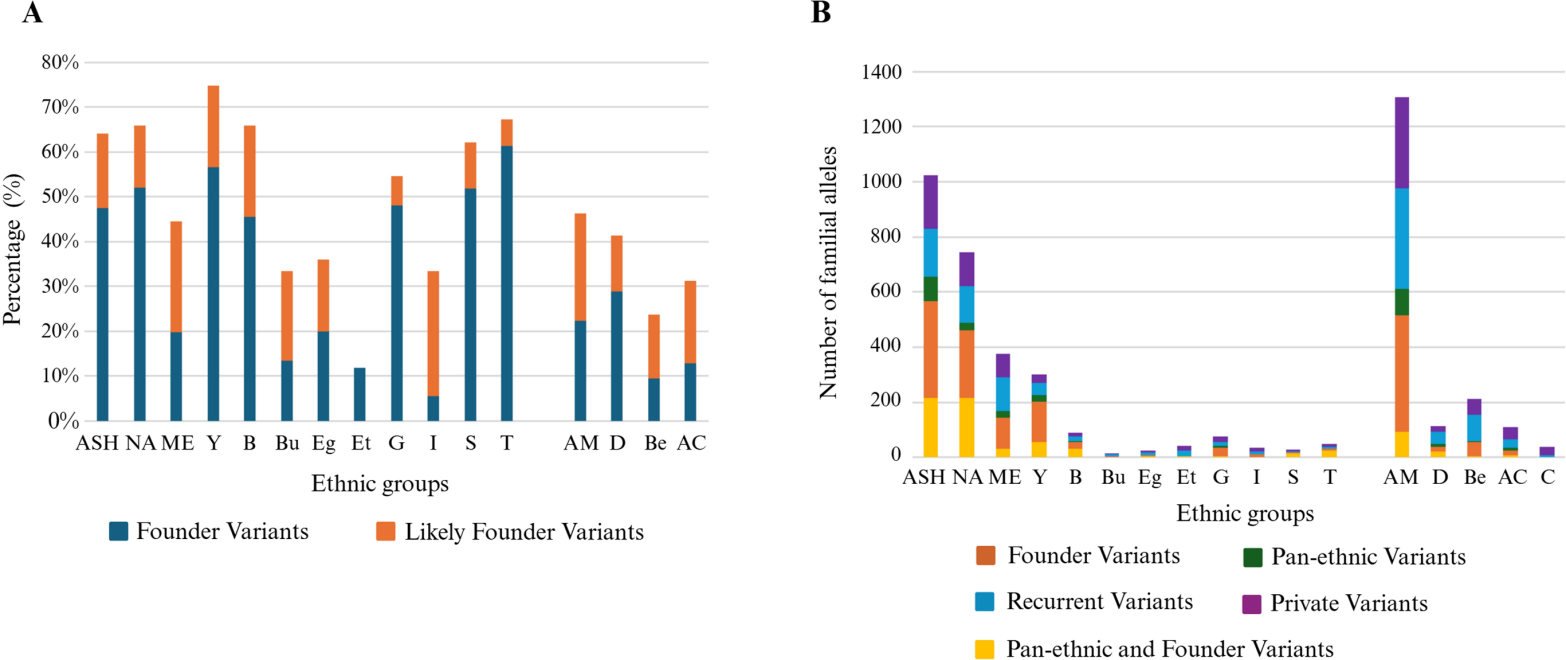
The percentage of founder variants per ethnic group **(A)** and the familial alleles count for each disease-causing variant type per ethnic group **(B)**. For each group the effect of five different types of variants is shown: founder (*orange*), pan-ethnic (*green*), recurrent (*blue*), private (*purple*), and both pan-ethnic and founder (*yellow*). AC, Arab Christian; AM, Arab Muslim; B, Bukhara; Be, Bedouin; Bu, Bulgaria; C, Christian; D, Druze; G, Georgia; I, India.

The percentage of founder and likely founder variants varies between different ethnic groups with the highest rate in Yemenite Jews (75%) followed by Turkish Jews (67%) and North-African Jews (66%) (Fig. 3A). Similarly, other types of disease-causing variants also vary between different ethnic groups; for example, pan-ethnic variants are most common in Arab Christians (10%) (Fig. 3B).

Although most of the Jewish disease-causing variants had a common haplotype among the various ethnic groups, one, *CRB1*-c.3307G>A, showed different haplotypes between Jewish (*n* = 7) and Muslim cases (*n* = 10), indicating that this variant arose independently. Another interesting disease-causing variant is *ABCA4*-c.5460+1G>A, which showed a shared haplotype between Arab-Muslim and Arab Christian.

### Phenotype Distribution

**Figure 4.**
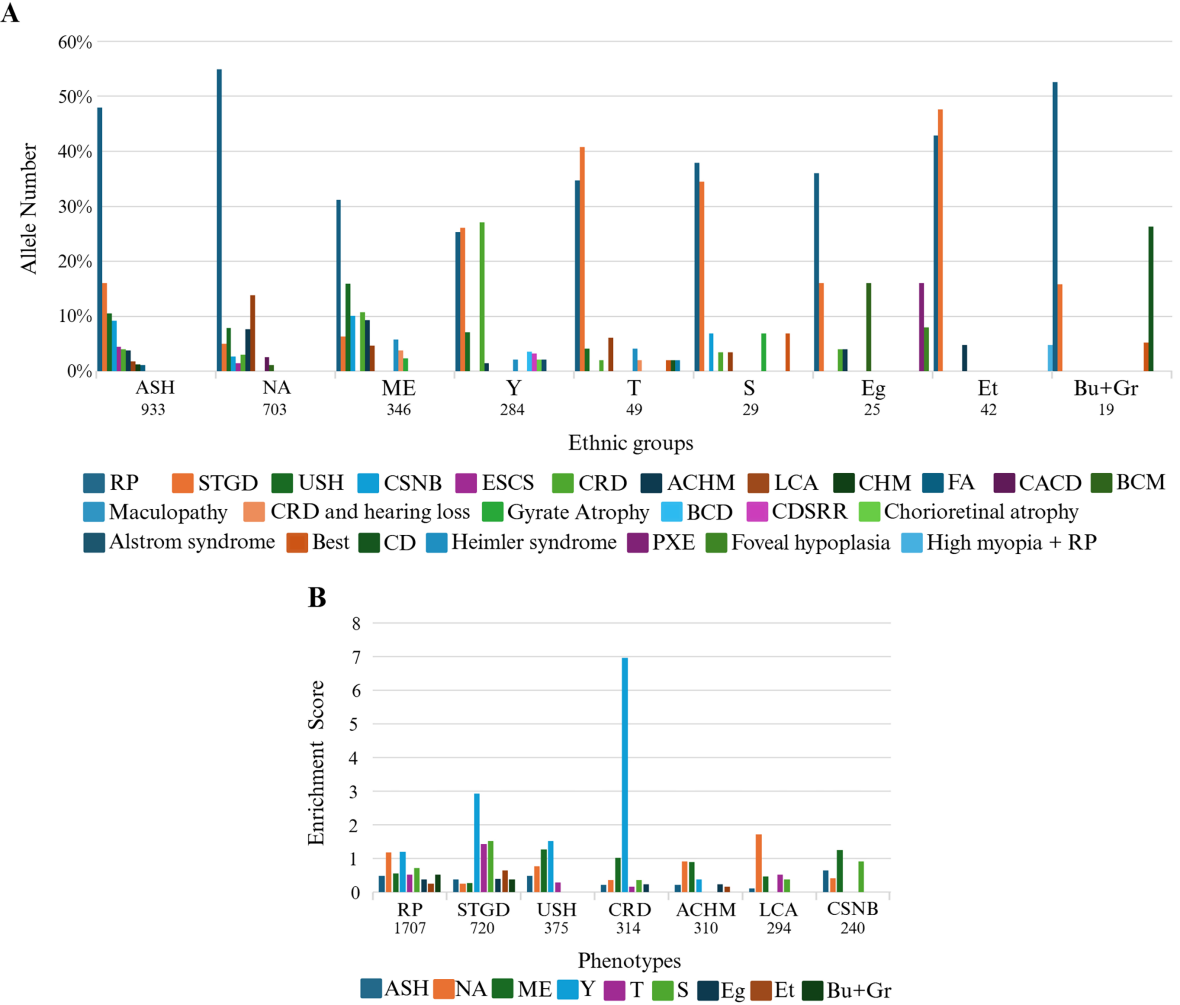
The 10 most common phenotypes for the main Jewish ethnic groups **(A)** and the correlation between the general population compared to our cohort for different phenotypes for the main Jewish ethnic groups **(B)**. ACHM, achromatopsia; BCD, Bietti crystalline dystrophy; BCM, blue-cone monochromacy; Bu, Bulgaria; CACD, central areolar choroidal dystrophy; CD, cone dystrophy; CDSRR, cone dystrophy with supernormal rod ERG; CHM, choroideremia; CRD, cone-rod dystrophy; CSNB, congenital stationary night blindness; ESCS, enhanced S-cone syndrome; FA, fundus albipunctatus; Gr, Greece; LCA, Leber congenital amaurosis; PXE, pseudoxanthoma elasticum; RP, retinitis pigmentosa; STGD, Stargardt disease; USH, Usher syndrome.

We subsequently studied the distribution of IRD phenotypes in the various ethnic groups (Fig. 4A). RP is the most common phenotype (55% in North-African Jews and 48% in Ashkenazi Jews). However, in some minor groups, such as Ethiopian Jews and Turkish Jews, Stargardt disease is the most common phenotype (48% and 41%, respectively). A few phenotypes have a relatively high prevalence in specific ethnic groups because of founder variants, as shown in Figure 4B, in which the enrichment score was calculated for the various phenotype-ethnic group combinations. For example, cone-rod dystrophy is especially common in Yemenite Jews (because of *CERKL*-c.238+1G>A) and Leber congenital amaurosis in North-African Jews (due to *RPE65*-c.95-2A>T and *GUCY2D*-c.389del; Fig. 4B).

**Figure 5.**
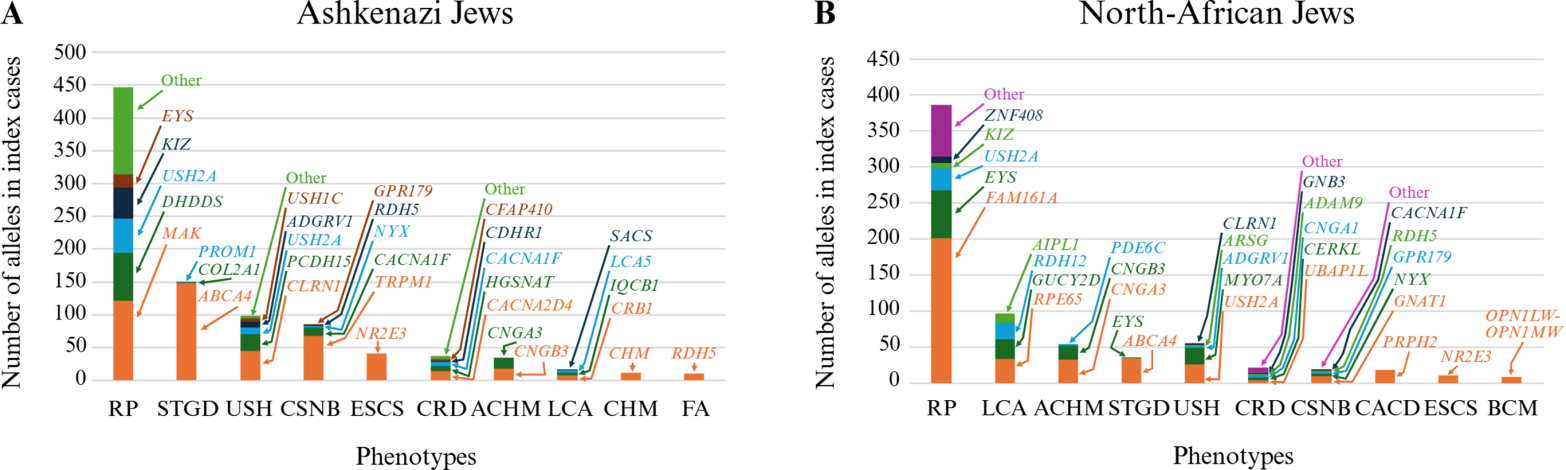
The five most common genes for the 10 most common phenotypes in Ashkenazi Jews **(A)** and North-African Jews **(B)**. Each phenotype is divided into the five most common genes. BCM, blue cone monochromacy.

The effect of founder variants on the distribution of IRD phenotypes is more evident in the most common ethnic groups, Ashkenazi Jews and North African Jews (Figs. 5A, 5B, respectively). In both ethnic groups, RP is the most common phenotype, however this is a result of different founder variants in *MAK* and *DHDDS* (causing 27% and 16% of RP cases in Ashkenazi Jews) and in *FAM161A* (causing 52% of RP cases in North-African Jews; Fig. 5). The analysis also highlights IRD monogenic phenotypes such as choroideremia in all groups.

### Variants Among the Jewish Population

As previously mentioned, some Jewish founder variants are specific only to one Jewish ethnic group while others are shared between multiple groups. To examine this in more details we studied disease-causing variants with at least 10 familial alleles that were found in at least three Jewish ethnic groups. Twelve disease-causing variants followed these criteria, and nine of them were specific only to Jews (e.g., *KIZ*-c.226C>T; Supplementary Fig. S6). In a previous study^22^ that identified this disease-causing variant haplotype analysis between two patients was performed, and a shared haplotype was observed.

### Variants in the Arab-Muslim Population

The Arab-Muslim sub-population is characterized by high rates of consanguinity and founder pathogenic variants that are specific to villages.^4^ In our cohort, 70% of the Arab-Muslim families reported consanguinity and the families are mostly spread in two distinct national regions: Jerusalem and the North (42% and 49% of all Muslim cases, respectively). In contrast, recruited Jewish families are relatively evenly spread across the country. Among the 29 most common Arab-Muslim pathogenic variants, 14 are specific to only a single region (and usually a specific village in that region).

For example, the most common disease-causing variant among Arab-Muslims is *TRPM1*-c.880A>T (p.K294*),^23^ with 44 familial alleles, all of which are only from Jerusalem area and most (73%) from a specific village (JM). Another common disease-causing variant is *PRCD*-c.64C>T (p.R72*), with 34 familial alleles in our cohort, all from a single village (EM) in the North region, as previously published.^24^

Although almost half of the disease-causing variants show region and village specificity, some variants are spread in multiple regions in Israel. For example, *ABCA4*-c.4979C>T has 20 familial alleles, is a founder variant with a shared haplotype between two Muslims from different regions in Israel (Jerusalem and the North).

### Generating an Online Database

Aiming to allow better access to the data presented here, we generated an online gene calculator tool (https://www.eyes.org.il/genecal), allowing the user to choose between three options: matching phenotype and ethnic group, matching gene and ethnic group and matching phenotype and gene. After the user choice selection, a pull-down menu appears for the selection of the gene, disease-causing variant, ethnic group, and phenotype of interest. The tool will calculate the frequency of the requested combination (An example for this can be found in Supplementary Fig. S7).

## Discussion

In the current study we characterized the ethnic-related genetic landscape of IRDs in the Israeli population, characterized by a relatively high number of founder variants due to the complex history of the Jewish people. Moreover, the relatively high rate of consanguinity may explain the high number of origin-specific disease-causing variants. In addition, an interesting group of disease-causing variants are those prevalent and distributed only (or mainly) among Jewish ethnic groups. These variants, including *FAM161A*-c.1355_1356del and *KIZ*-c.226C>T, have a shared haplotype among several different Jewish ethnic groups, further supporting the common origin of the Jewish people. As for the Muslim sub-population, most of the founder variants occur in specific villages such as the *PRCD*-c.64C>T and spread mainly through consanguinity. However, some disease-causing variants, such as the *ABCA4*-c.4979C>T, have a shared haplotype among Muslims from different regions in Israel.

In the current manuscript we used an updated version of the IIRDC cohort including 2676 families. Two previous publications^4^^,^^19^ are based on a previous version of this cohort and published results that show some similarity with the current study. However, in the current study we used additional methodology including WGS. For example, Ehrenberg et al^4^ reported that AR was the most common inheritance pattern and that 37% of the recruited families reported consanguinity. However, when comparing to the general Israeli population they found that for Jews and Druze the percentage in the cohort was lower than those reported in the general population. We, however, report here a similar result for the Jewish ethnic group but not for the Druze. The five most common causing genes identified here are identical to those reported by us previously^19^; however, their frequencies and the most common pathogenic variants identified in these genes were slightly different. Those differences could be due to additional families included in the current study, allowing more accurate conclusions.

The genetic landscape of IRDs can vary between different populations worldwide, corresponding to the population and their unique structure. Comparison to worldwide information was made by comparing the study cohort with a recent publication by the Foundation Fighting Blindness.^25^ This publication combined data from 30 sites located in the Unites States, Europe, Brazil and Israel. In the United States and Europe, the top five IRD-causing genes are *ABCA4* (16% and 18%, respectively), *USH2A* (9% for both), *RPGR* (7% and 5%), *PRPH2* (5% for both), and *RHO* (5% and 4%). The distribution in our cohort was somewhat different, starting with *ABCA4* (15%) and *USH2A* (7%), followed by *FAM161A* (5%), *CNGA3* (5%), and *EYS* (4%).

The genetic landscape of IRDs was previously reported in other populations^26^^–^^37^ (Table), including cohorts ranging from 105 to 3953 genetically solved index cases. In most of the studies, *ABCA4* was among the five most common genes, except for studies form Iran^35^ and Northern Finland.^33^

**Table.** Genetic Landscape Publications

Country/Population	Reference	Index Cases (Genetically Solved)	Year	The Five Most Common Genes
UK	Lin et al.^27^	3953	2024	*ABCA4, USH2A, RPGR, PRPH2, BEST1*
Spain	Perea-Romero et al.^28^	2100	2021	*ABCA4, USH2A, RS1, CRB1, RHO*
Arabs	Jaffal et al.^29^	1621	2023	*TULP1, ABCA4, RP1, CRB1, MYO7A*
Germany	Weisschuh et al.^26^	1250	2020	*ABCA4, USH2A, RPGR, RHO, PRPF31*
United States	Stone et al.^37^	760	2017	*ABCA4, USH2A, RPGR, RHO,PRPH2*
Argentina	Schlottmann et al.^30^	379	2023	*USH2A, RPGR, ABCA4, RHO, PRPF31* and *EYS*
Northern Finland	Lähteenoja et al.^33^	210	2025	*FZD4, RPGR, CHM, RS1, CERKL*
Portugal	Peter et al.^36^	174	2023	*EYS, ABCA4, RPGR, USH2A, RHO*
Pakistan	Ullah et al.^34^	171	2025	*ABCA4, CRB1, MYO7A, PDE6B, RP1*
Welsh	Sanders et al.^31^	166	2025	*ABCA4, USH2A, GUCY2D, RHO, PRPH2*
Iran	Ardehaie et al.^35^	133	2025	*CNGA3, AIPL1, TMEM67, BBS2, ADGRV1*
Mexico	Villanueva-Mendoza et al.^32^	105	2021	*ABCA4, CRB1, RPGR, USH2A, ARL6*

Some of the articles also reported on the most common inheritance pattern^26^^,^^29^^,^^30^^,^^33^^–^^37^; for the majority of studies AR was the most common, ranging from 45% in Northern Finland to 96% in Pakistan, compared to 82% in the current study. The high rate of AR inheritance in specific cohorts such as Pakistan and Iran can be due to the extremely high rate of consanguinity (86% in the Iranian population), as well as geographical isolation and founder variants.

In contrast to most of these studies, which represent only a relatively small number of the IRD cases in each country, the data presented here are based on a nationwide analysis allowing us to study gene and disease-causing variant distribution among sub-populations. This is one of the largest cohorts, which enables us to give a wide view of the genetic landscape in Israel.

As for pan-ethnic variants, the most interesting one is *ABCA4*-c.5882G>A. This is the most prevalent disease-causing variant in the studied cohort, was previously reported to be prevalent across different populations, and was observed across all the major gnomAD populations. Interestingly, *ABCA4*-c.5882G>A showed a shared haplotype between multiple Jewish ethnic groups and Arab-Muslims, indicating that this allele is ancient and shared by origin between multiple populations.

This study has a few limitations that should be pointed out. First, some affected individuals may have been recruited by more than one center, leading to an overestimation. In addition, siblings who belong to the same family might have been recruited as separated ones. However, this is at least partially overcome by the fact that consortium members regularly interact to identify such cases and to ensure that genetic analysis is performed by a single center. Second, although efforts were made to unify diagnostic criteria and data collection protocols across centers, some variability in clinical assessment and data annotation may be present, potentially affecting the uniformity of phenotype-genotype correlations. Third, Figure 1 depicts enrichment scores of the various subpopulations, and the population data is based on the Israeli central bureau of statistics, which combined data of Arab-Christians and non-Arab Christians into one group. We therefore can't perform a separate analysis for Christians alone. We do predict that such analysis will show lower enrichment level for Christians because of lower consanguinity rates.

## Conclusions

This study provides the genetic landscape of IRDs in the Israeli population. The data presented here highlight what are the most common disease-causing variants in each ethnic group in the Israeli population and direct the development of therapeutic modalities targeting those variants. Furthermore, creating a database can help organize and make the data more accessible for patients, genetic counselors and other health care professionals. Obtaining similar results from cohorts in other countries will allow a more comprehensive understanding of the genetic landscape of IRDs and will allow the scientific community to better direct available gene- and variant-specific therapies to the appropriate population.

## Supplementary Material

Supplement 1

Supplement 2
