# Supplementary material for: The Genetic Landscape of Inherited Retinal Diseases in the Israeli Population
Source: Invest Ophthalmol Vis Sci. 2026 Apr 13;67(4):24. doi: 10.1167/iovs.67.4.24 (PMC13089656; doi:10.1167/iovs.67.4.24)
Supplement: Supplement 1 [file iovs-67-4-24_s001.docx]

SUPPLEMENTAL MATERIAL

The genetic landscape of inherited retinal diseases in the Israeli population

Sapir Shalom BSc^1,2^, Libe Gradstein MD^3^, Eran Pras MD^4^, Johanna Valensi MSc^1^, Ohad S. Birk MD, PhD^5^, Anat Blumenfeld PhD^1^, Avital Eilat MSc^1^, Michal Macarov PhD^1^, Tomer Poleg BSc^5^, [Frans P M Cremers](https://pubmed.ncbi.nlm.nih.gov/?sort=date&term=Cremers+FPM&cauthor_id=31456290) PhD^6^, [Susanne](https://pubmed.ncbi.nlm.nih.gov/?sort=date&term=Cremers+FPM&cauthor_id=31456290) Roosing PhD^6^, [Daan](https://pubmed.ncbi.nlm.nih.gov/?sort=date&term=Khan+MI&cauthor_id=31456290) Panneman PhD^6^, Nadin Hollander^7^, Nitza Goldenberg-Cohen MD^8,9^, Claudia Yahalom MD^1^, Eyal Banin MD, PhD^1^, Tamar Ben-Yosef PhD ^9^, Dror Sharon PhD^1^

1- Department of Ophthalmology, Hadassah Medical Center, Faculty of Medicine, The Hebrew University of Jerusalem, Jerusalem.

2- Department of Military Medicine and "Tzameret", Faculty of Medicine, Hebrew University of Jerusalem and Medical Corps, Israel Defense Forces, Jerusalem, Israel.

3- Department of Ophthalmology, Soroka Medical Center and Clalit Health Services, Faculty of Health Sciences, Ben‐Gurion University, 84101 Beer Sheva, Israel.

4- Faculty of medicine, Tel Aviv university, Tel Aviv, Israel.

5- Faculty of Health Sciences at Ben Gurion University of the Negev, Genetics Institute at Soroka Medical Center, and the Danek Gertner Institute of Human Genetics at Sheba Medical Center, Ramat Gan, Israel.

6- Department of Human Genetics, Radboud University Medical Center, Nijmegen, The Netherlands.

7- The “Lirot” association, Tel Aviv, Israel.

8- Department of Ophthalmology, Bnai-Zion Medical Center, Haifa, Israel.

9- Rappaport Faculty of Medicine, Technion - Israel Institute of Technology, Haifa, Israel.

Corresponding author:

Dror Sharon, PhD., Department of Ophthalmology, Hadassah-Hebrew University Medical Center, Jerusalem, Israel, 91120; Phone: +972 2 6777112; Fax: +972 2 6448917; E-mail: [dror.sharon1@mail.huji.ac.il](mailto:dror.sharon1@mail.huji.ac.il)

**Supplementary Methods**

**Genetic analysis**

Genetic analysis was performed in each IIRDC center and included sanger sequencing, panel sequencing, whole exon sequencing (WES) and whole genome sequencing (WGS). Different panels were used over the years such as molecular inversion probes (MIPs) panels, founder variants panels and more. For each patient different sequencing method was chosen based on clinical estimation and ethnic group. Some samples were sequenced in various methods. File conversion was preformed using Galaxy platform (<https://usegalaxy.org/>) and Franklin (<https://franklin.genoox.com/clinical-db/home>). Data annotation and filtering were done mainly using the Franklin platform by filtering the data using an IRD gene panel. Variants were filtered based on minor allele frequence (MAF) from the gnomAD dataset (<https://gnomad.broadinstitute.org/>).

Each variant was scored for pathogenicity based on current available information, including ClinVar entries, gnomAD frequency data, cohort frequency data, online pathogenicity scoring tools, and familial segregation analyses. Variants with no previous classification that were common in our patient cohort, or/and had functional predictions, or were in trans with null variants were added to Franklin database and classified as likely pathogenic. Each unclassified variant was verified based on patient's phenotype, family segregation and additional data before considering as disease-causing. Most of the unclassified variants are founder or private variants that can be found mainly in our cohort and therefore were not reported in the past.

**Enrichment score**

We used the enrichment score calculation to examine whether a specific ethnic group was enriched in the cohort compared to the general population according to the available data from the central bureau of statistics. The Bulgarian and Greek Jewish ethnic groups, as well as the Arab-Christian and Christians groups were combined to match the data from the central bureau of statistics. The percentage of alleles from each ethnic group in our cohort was calculated by dividing the number of familial alleles for each ethnic group by the total number of familial alleles per index cases. The percentage of each ethnic group in the Israeli population was calculated by dividing the number of people in each group in the population by the population size as of September 2023. The enrichment score was obtained by dividing the percentage of the participants from each ethnic group in our cohort by the percentage of people from the same group in the population according to the central bureau of statistics. A score >1 indicates that the number of affected individuals in the cohort of IRD patients is above the expected value based on the fraction of this ethnic group in the population.

**Heat map calculation**

To gain a better understanding of disease-causing variants distribution across various populations, we focused on the 319 most frequent variants. For each disease-causing variant, we tabulated the number of reported index cases per ethnic group. The data were analyzed in two complementary approaches: variant-based and origin-based. In the variant-based analysis, we calculated the proportion of index cases from a given origin relative to the total number of individuals carrying the same disease-causing variant across all ethnic groups. This yields a ratio between 0 and 1, where a value of 1 indicates that all index cases with a given disease-causing variant originate from the same population. In the origin-based analysis, we computed the proportion of index cases in each ethnic group who carry a specific disease-causing variant. This was done by dividing the number of index cases with a given variant by the total number of cases reported from that origin, again yielding a frequency between 0 and 1. A value of 1 here means that all affected individuals from that origin carry the same disease-causing variant.

**Figure S1**: The most common mutated genes (**A**) and disease-causing variants (**B**) in the studied cohort.


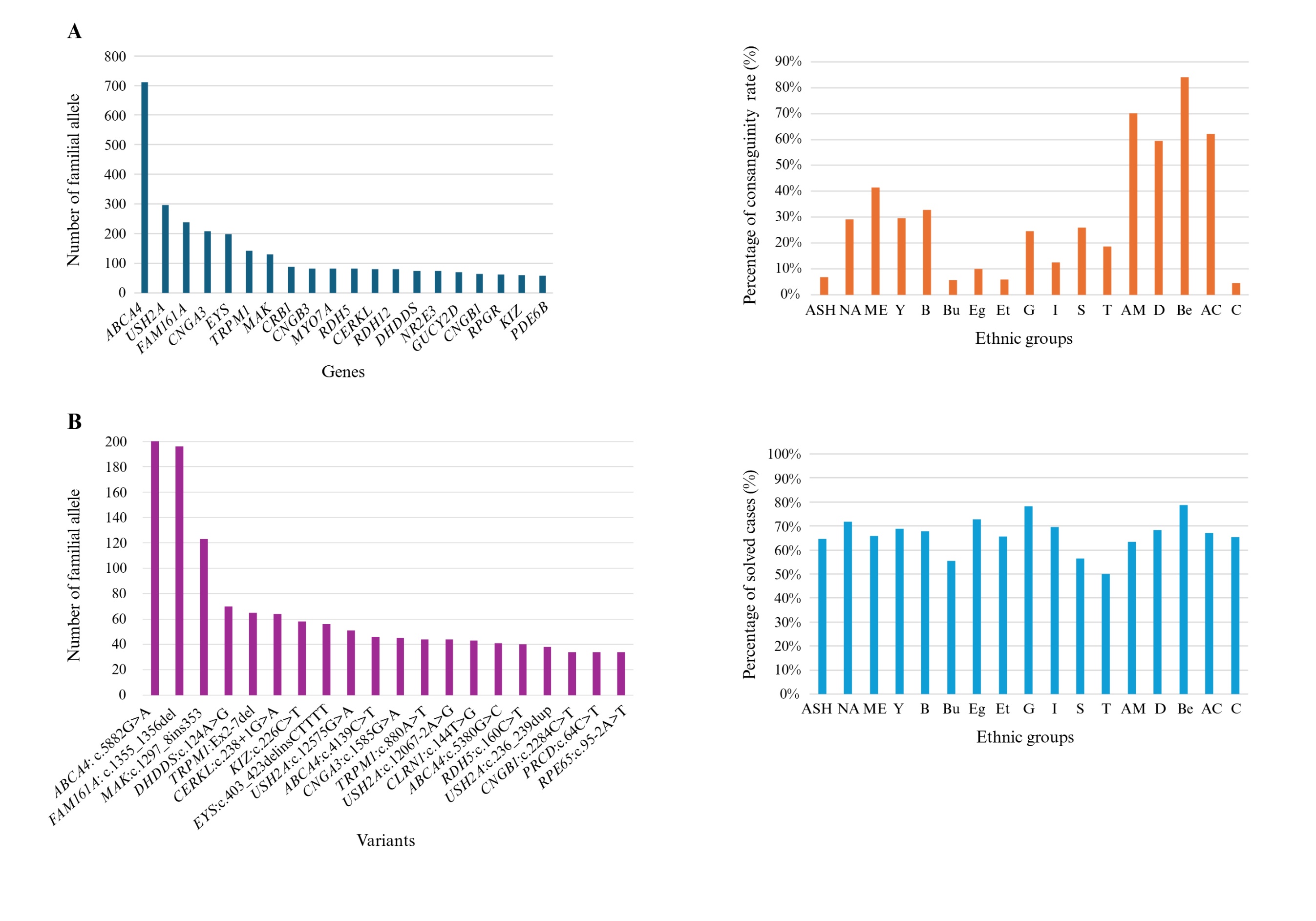


**Figure S2**: The rate of consanguinity in each ethnic group in the studied cohort.

**
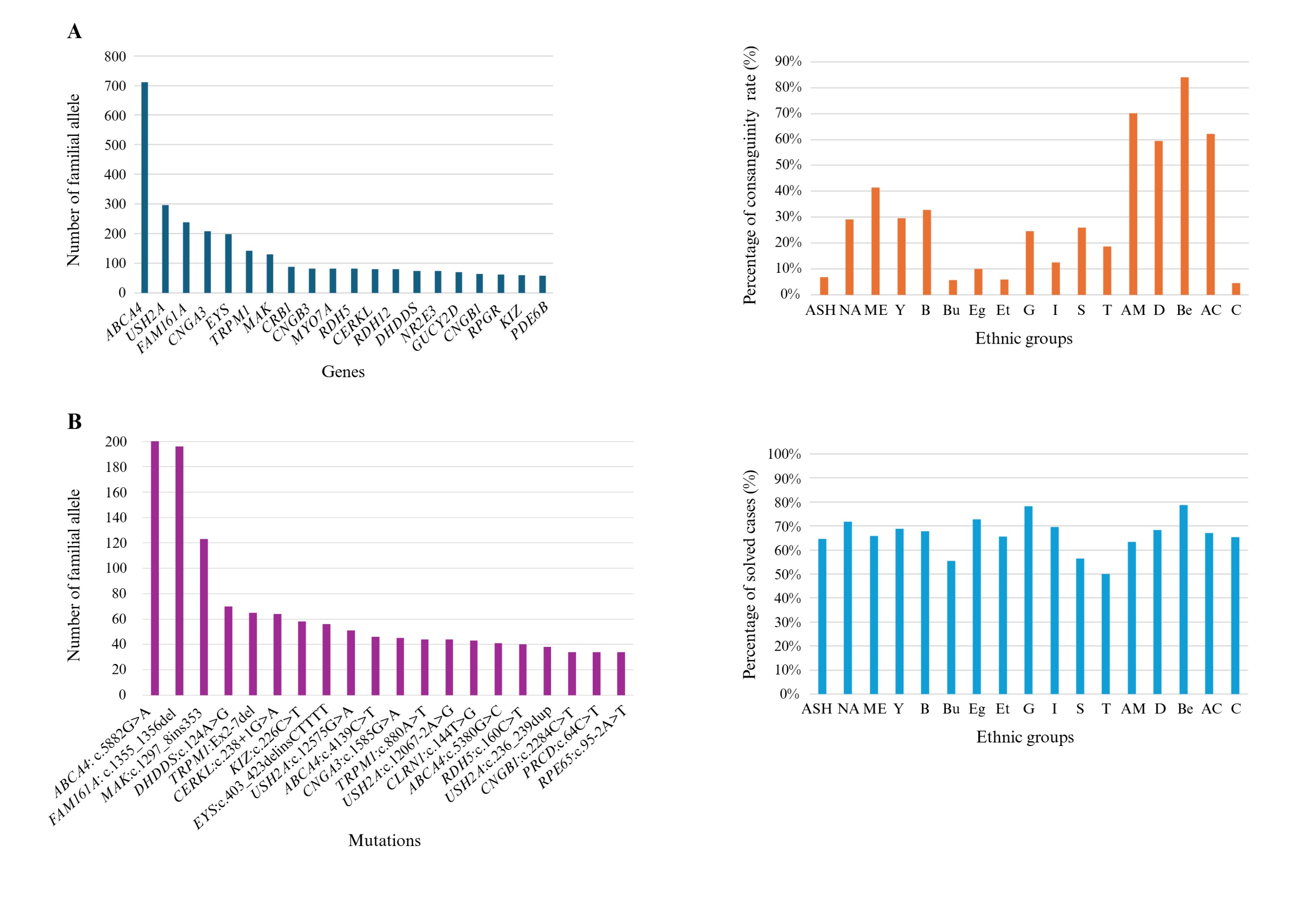
**

**Abbreviation:** ASH- Ashkenazi, NA- North African, ME- Middle Eastern, Y- Yemen, B- Bukhara, Bu- Bulgaria, Eg- Egypt, Et- Ethiopia, G- Georgia, I- India, S- Syria, T- Turkey, AM- Arab Muslim, D- Druze, Be- Bedouin, AC- Arab Christian**,** C**-** Christian**.**

**Figure S3:** The percentage of solved patients in each ethnic group in the studied cohort.


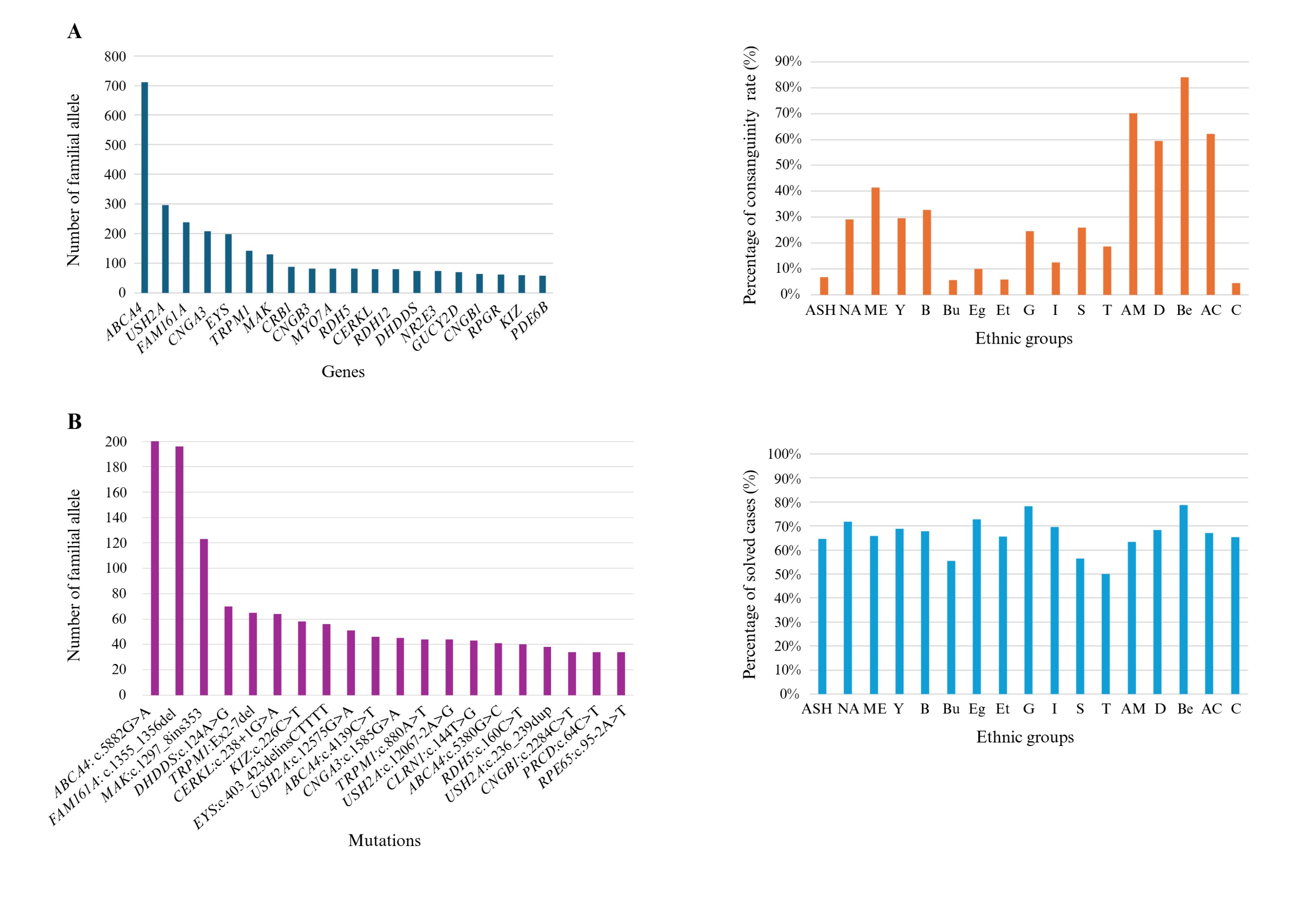


**Abbreviation:** ASH- Ashkenazi, NA- North African, ME- Middle Eastern, Y- Yemen, B- Bukhara, Bu- Bulgaria, Eg- Egypt, Et- Ethiopia, G- Georgia, I- India, S- Syria, T- Turkey, AM- Arab Muslim, D- Druze, Be- Bedouin, AC- Arab Christian**,** C**-** Christian**.**

**Figure S4:** The inheritance pattern (**A and B**) and Zygosity (**C and D**) in each ethnic group in the studied cohort.

**
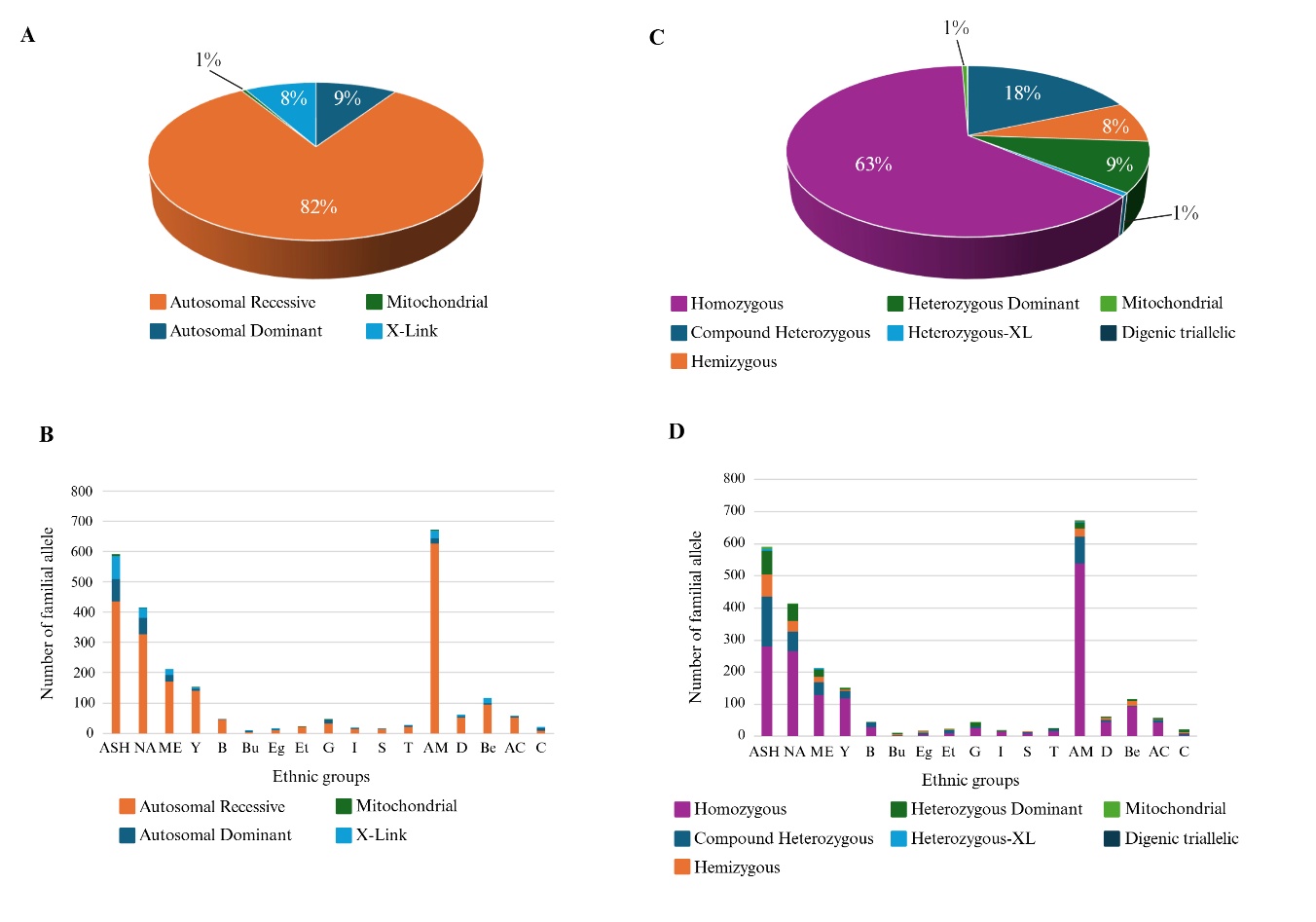
Abbreviation:** ASH- Ashkenazi, NA- North African, ME- Middle Eastern, Y- Yemen, B- Bukhara, Bu- Bulgaria, Eg- Egypt, Et- Ethiopia, G- Georgia, I- India, S- Syria, T- Turkey, AM- Arab Muslim, D- Druze, Be- Bedouin, AC- Arab Christian**,** C**-** Christian**.**

**Figure S5:** The genomic distribution of founder variants.

**
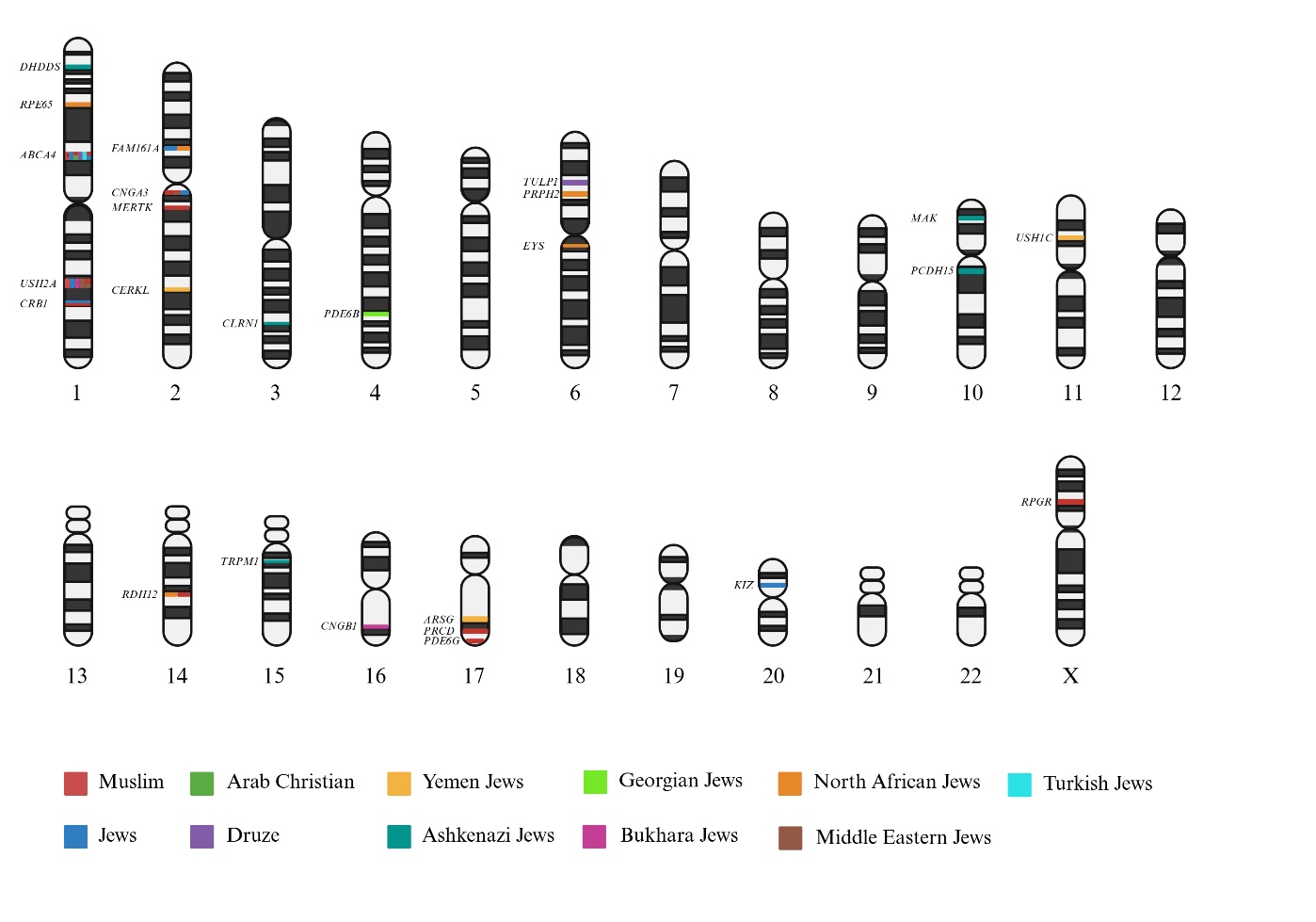
Figure legends:** The figure is color-coded by the various ethnic groups. For genes with more than one founder variant a few colors are presented next to each other. For disease-causing variants that appeared in more than one ethnic group two colors are presented one on top of the other. If an ethnic group has more than one disease-causing variant in the same gene a number is written on this group's color and represents the number of variants.

**Figure S6:** Haplotype analysis for the *KIZ*- c.226C>T variant in the Jewish population.


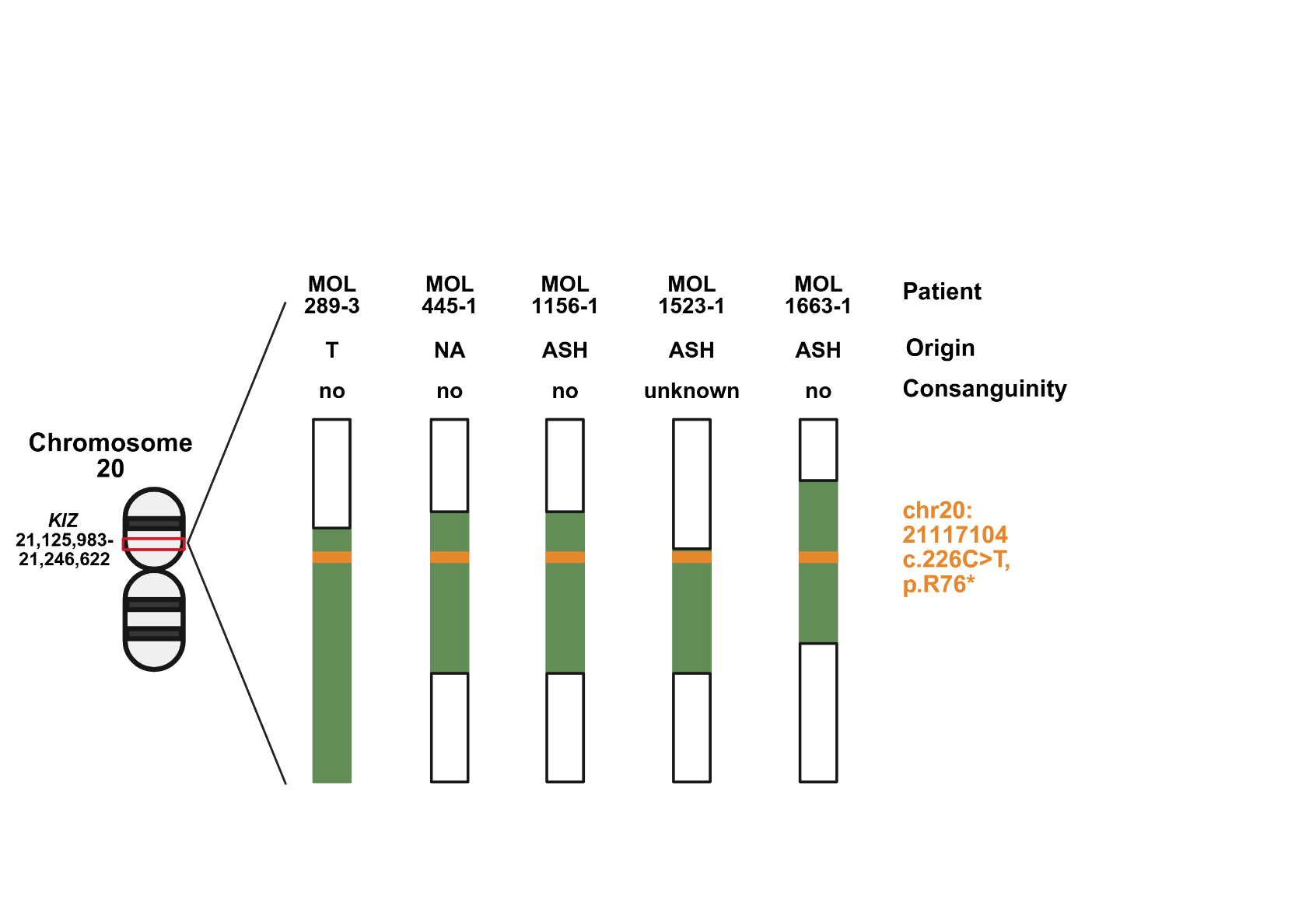


**Figure legends:** This disease-causing variant was found in seven different Jewish ethnic groups and showed a shared haplotype between three ethnic groups (Turkish, North-African and Ashkenazi Jews; NGS data was not available for the remaining four groups). Orange line represents the pathogenic variant location; green represents the shared homozygous haplotype and white represents the regions where the homozygous shared haplotype is disrupted. **Abbreviation:** ASH- Ashkenazi, NA- North African, T- Turkish.


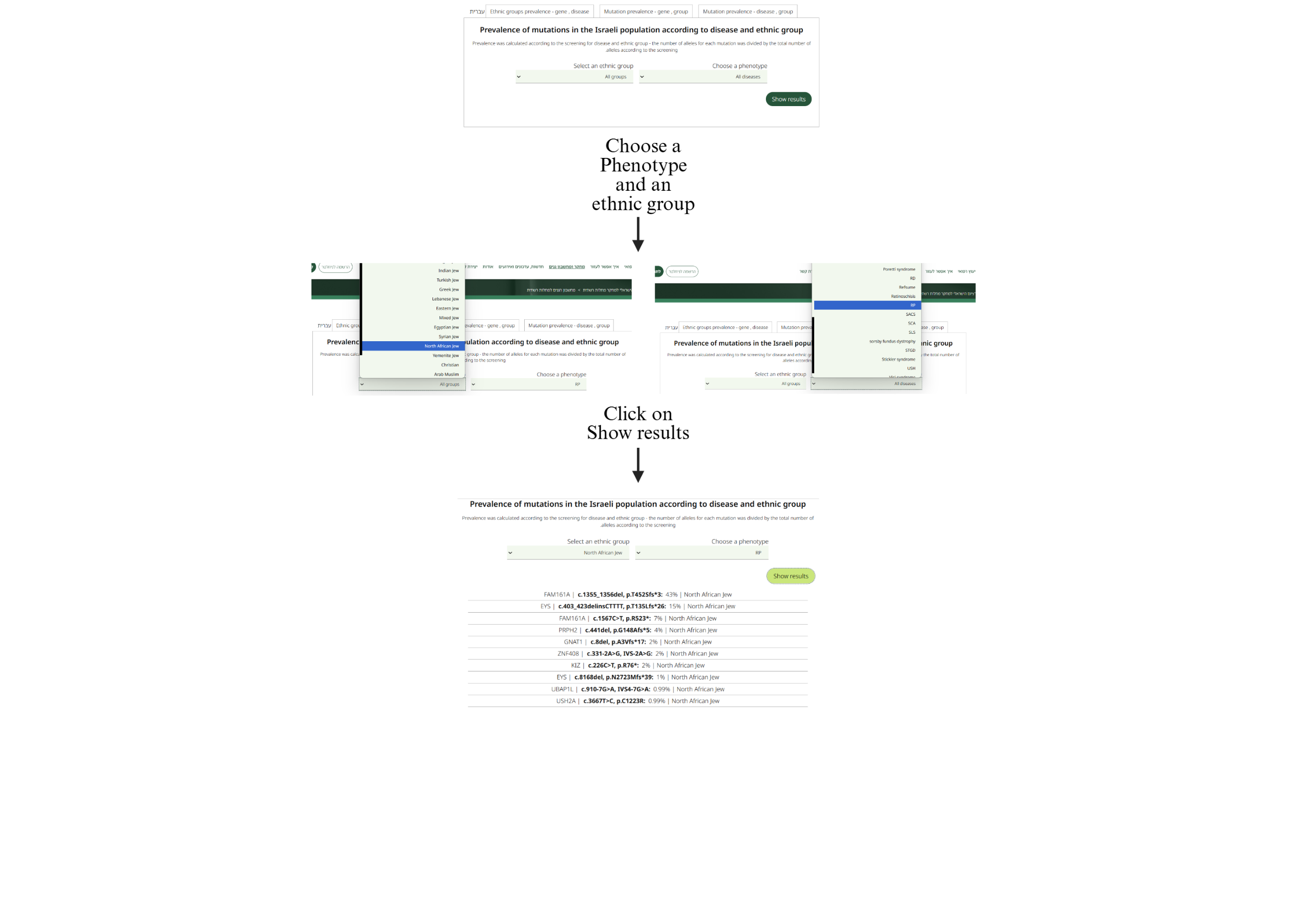
**Figure S7:** An example for using the gene calculator toll.
